# The Frequency of Convergent Games under Best-Response Dynamics

**DOI:** 10.1007/s13235-021-00401-3

**Published:** 2021-10-19

**Authors:** Samuel C. Wiese, Torsten Heinrich

**Affiliations:** 1 Department of Computer Science, University of Oxford, Oxford, OX1 3QD UK; 2 Institute for New Economic Thinking, University of Oxford, Oxford, OX1 3UQ UK; 3 Department of Economics and Business Administration, Chemnitz University of Technology, 09107 Chemnitz, Germany; 4 Oxford Martin School, University of Oxford, Oxford, OX1 3BD UK

**Keywords:** Pure Nash equilibrium, Best-response dynamics, Random games, 91A10, 91A06

## Abstract

We calculate the frequency of games with a unique pure strategy Nash equilibrium in the ensemble of *n*-player, *m*-strategy normal-form games. To obtain the ensemble, we generate payoff matrices at random. Games with a unique pure strategy Nash equilibrium converge to the Nash equilibrium. We then consider a wider class of games that converge under a best-response dynamic, in which each player chooses their optimal pure strategy successively. We show that the frequency of convergent games with a given number of pure Nash equilibria goes to zero as the number of players or the number of strategies goes to infinity. In the 2-player case, we show that for large games with at least 10 strategies, convergent games with multiple pure strategy Nash equilibria are more likely than games with a unique Nash equilibrium. Our novel approach uses an *n*-partite graph to describe games.

## Introduction

A Nash equilibrium in a normal-form game is a strategy profile such that, given the choice of the other players, no player has an incentive to make a different choice. If the Nash equilibrium is in pure strategies, we call it *pure strategy Nash equilibrium* (PSNE), otherwise *mixed strategy Nash equilibrium* (MSNE). John Nash showed that any game with a finite number of players and strategies has at least one MSNE (Nash [15, 16]). This is not the case for PSNEs.

Consider an *n*-player, *m*-strategy normal-form game and assume that players choose their optimal strategy (facing previous optimal strategies of the opponents) in a clockwork sequence—player 1 goes first, then player 2, etc. until its player 1’s turn again. We call a game *convergent*, if starting from any initial strategy profile no player changes their strategy under the described dynamic after a sufficiently large number of turns.

We describe such games by an *n*-partite graph with each node corresponding to a pure strategy profile of the strategy choices of all but one player, and each edge corresponding to the optimal strategy choice (*best response*). A PSNE corresponds to a shortest possible cycle of length *n*.

In general, there are three types of games:

- Type A: Convergent games with a unique PSNE
- Type B: Convergent games with multiple PSNEs
- Type C: Non-convergent games

Type A games (for instance, the Prisoners’ Dilemma) are very easy to understand and perfectly predictable. They converge to the PSNE. As we may re-arrange the strategies of the players, Type B games are coordination games. An example of a Type C game is Matching Pennies. Type B and Type C games have at least one MSNE.

We will investigate the likelihood of randomly created games that converge (Type A and Type B) in the ensemble of games with a given number of players and a given number of strategies available to each player. The frequencies can provide insights into predictability and stability of equilibria in economic systems. For situations that are conveniently modelled by low-dimensional (e.g. 2-player 2-strategy) games, predictability and stability properties are often obvious. For more complicated biological interactions [8, 9], bidding behaviour [5], interactions on supply chains [3], trading behaviour in financial markets [4], or social behaviour during a crisis (say the COVID-19 pandemic), this is different.

We will show that Type A and Type B games become less likely the more complex the game is. Thus, modelling scenarios like climate change or financial market events with Type A or Type B games would lead to misleading results. In spite of the involved difficulty, it would be expedient to employ models that use Type C games.

### Related Work

Several papers have considered aspects related to the number of PSNE in games with random payoffs. We briefly consider the papers that dealt with random payoffs that are i.i.d. from a continuous distribution.

Goldman [11] considered zero-sum 2-player games and showed that the probability of having a PSNE goes to zero as the number of strategies grows. Goldberg et al. [10] considered general 2-player games and showed that the probability of having at least one PSNE converges to $1-\exp (-1)$ as the number of strategies goes to infinity. Dresher [6] generalized this result to the case of an arbitrary finite number of players. Powers [19] showed that, when the number of strategies of at least two players goes to infinity, the distribution of the number of PSNEs converges to Poisson(1). Stanford [20] derived an exact formula for the distribution of the number of PSNEs in random games, and showed that for two-person symmetric games, the number of symmetric and asymmetric PSNEs converges to a Poisson distribution [21]. McLennan [13] obtained a computationally implementable formula for the mean number of Nash equilibria.

Alon et al. [1] studied the frequency of dominance-solvable games and obtained an exact formula for the 2-player case. Dominance-solvable games are necessarily convergent, but not vice versa, so we study a larger class of games (containing, for instance, coordination games). The unique PSNE in Type A games are called *Cournot stable*; this class of games was studied by Moulin [14].

Concerning the use of best-response structures as a tool to study convergence frequencies, Pangallo et al. [17] and Pei and Takahaski [18] both obtained exact results for the frequency of one or more PSNEs in the 2-player case. The authors in [12] use different methods to bound the convergence frequency in multi-player games.

Finally, random games were studied in the context of theoretical biology, for instance, the authors in [7] investigated the distribution of equilibria of an evolutionary dynamic.

### Our Contribution

We introduce an *n*-partite graph describing the best responses of a game and use it to obtain the frequency of randomly created games with a unique PSNE in the ensemble of *n*-player, *m*-strategy games. These games are perfectly predictable. We then study games with more than one PSNE, that are convergent under best-response dynamics, in which each player successively chooses their optimal pure strategy. We show that convergent games with a smaller number of PSNEs are more common than convergent games with a higher number of PSNEs. We obtain an exact frequency for convergent 2-player games with any given number of PSNEs. We finally highlight that for 2 players and less than 10 strategies, games with a unique PSNE are more common than convergent games with multiple PSNEs, otherwise less common.

## Methods

### Notation

A game with $n\ge 2$ players and $m\ge 2$ strategies available to each player is a tuple $(N,M,\{u_i\}_{i\in N})$ where $N=\{1,\dots ,n\}$ is the set of players, $M=\{1,\dots ,m\}$ the set of strategies for each player, and $u_i:M^n\rightarrow {\mathbb {R}}$ a payoff function. A *strategy profile* $s=(s_1,\dots ,s_n)\in M^n$ is a set of strategies for each player. An *environment* for player *i* is a set $s_{-i}\in M^{n-1}$ of strategies chosen by each player but *i*. A *best response* $b_i$ for player *i* is a mapping from the set of environments of *i* to the set of non-empty subsets of *i*’s strategies and is defined by

$$\begin{aligned} b_i(s_{-i}) := {{\,\mathrm{arg\,max}\,}}_{s_i\in M} u_i\left( s_i,s_{-i}\right) . \end{aligned}$$

A strategy profile $s\in M^n$ is a *pure strategy Nash equilibrium* (PSNE) if for all $i\in N$ and all $s_i\in M$,

$$\begin{aligned} u_i(s) \ge u_i(s_i,s_{-i}). \end{aligned}$$

Equivalently, $s\in M^n$ is a PSNE if for all $i\in N$ and all $s_i\in M$, $s_i\in b_i(s_{-i})$. A game is *non-degenerate*, if for each player *i* and environment $s_{-i}$, the best-response $b_i(s_{-i})$ is a singleton; we then write $s_i=b_i(s_{-i})$. Similarly, a *mixed strategy Nash equilibrium* (MSNE) is a strategy profile in mixed strategies.

### Games as Graphs

The best-response structure of a game can be represented with a best-response digraph whose vertex set is the set of strategy profiles $M^n$ and whose edges are constructed as follows: for each $i\in N$ and each pair of distinct vertices $s=(s_i,s_{-i})$ and $s'=(s'_i,s_{-i})$, place a directed edge from *s* to $s'$ if and only if $s'_i=b_i(s_{-i})$. There are edges only between strategy profiles that differ in exactly one coordinate.

We now introduce an *n*-*partite graph* as an additional representation of the best responses for a given fixed sequence of players. There is a total of $nm^{n-1}$ nodes in *n* groups, each group corresponding to a player and each node corresponding to an environment of a player. At each node, a player chooses the best response; formally, the edges are constructed as follows: for each pair (*i*, *j*) of players, where *j* moves directly after *i*, and each environment $s_{-i}=\left( s_j,s_{-i,-j}\right)$ (where $s_{-i,-j}$ is $s_{-i}$ without the strategy choice of *j*), place a directed edge from $s_{-i}$ to another environment $s'_{-j}=(s'_i,s_{-i,-j})$, if and only if

⋆ $$\begin{aligned} s'_i=b_i(s_{-i}). \end{aligned}$$

As we can assume that games are non-degenerate, each node in a graph representing a game has an out-degree of 1. A PSNE corresponds to a cycle of length *n*. Each player chooses among *m* strategies at each node, thereby the total number of possible arrangements is $m^{nm^{n-1}}$, each equally likely.

We call the *n*-partite graph constructed as above but without the condition (⋆) the *full n-partite graph* (see Fig. 5 (left)). Any *n*-partite graph corresponding to a given game is a subgraph of the full *n*-partite graph. We will call a node *free*, if its out-degree is *m*, and *fixed*, if its out-degree is 1.

**Fig. 1:**
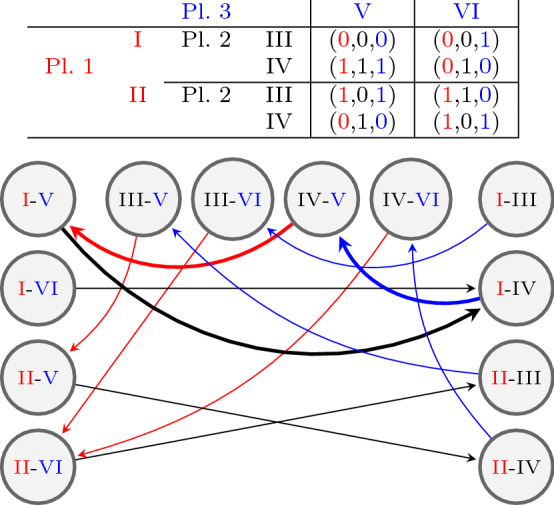
A 3-player, 2-strategy game with one PSNE and the corresponding 3-partite graph representation. The best responses corresponding to the PSNE (I–IV–V) are highlighted

Figure 1 shows a 3-player, 2-strategy game with one PSNE and the corresponding 3-partite graph with playing sequence 1-2-3.

We could have relaxed the assumption of assuming only one specific sequence of players. However, PSNEs are stable under any playing sequence. Introducing random or other playing sequences would make the digraph more complicated by adding more arcs without changing the results. Dynamics where players choose their strategies simultaneously cannot be represented by an n-partite graph.

## Results

### Type A: Convergent Games with a Unique PSNE

We generate *n*-player, *m*-strategy games at random by drawing $m^n$ tuples of payoffs from a continuous distribution. This ensures that randomly created games are almost surely non-degenerate. The exact type of distribution does not matter as long as payoffs are uncorrelated, because the structure of the game only depends on which one in a set of values is the largest. We leave games with correlated payoffs for future research, but results from the literature ( [17]) suggest that Type A games are less likely when payoffs are negatively correlated and that Type B games (see below) become overwhelmingly likely with positive correlations.

Let $p_{n,m}^k$ denote the frequency of *n*-player, *m*-strategy convergent games with exactly *k* PSNEs.

#### Theorem 1

The frequency of games with one unique PSNE in the ensemble is given by

$$\begin{aligned} p_{n,m}^1 = r^{n-1}+\frac{m-1}{m-r} \left( \left( \frac{r}{m}\right) ^{n-1}-1\right) \end{aligned}$$

where $$r:=\frac{m-1}{m^n}+1$$.

Note, that the frequency $p_{n,m}^1\rightarrow 0$ as the number of strategies or the number of players goes to infinity, and that $p_{n,m}^1$ is decreasing in both *n* and *m*. For instance:

$$\begin{aligned} p_{2,m}^1 ={}&\frac{1}{m}\left( 2-\frac{1}{m}\right) \\ p_{3,m}^1 ={}&\frac{1}{m^2}\left( 3 - \frac{3}{m} + \frac{3}{m^2} - \frac{3}{m^3} + \frac{1}{m^4}\right) \\ p_{4,m}^1 ={}&\frac{1}{m^3}\left( 4 - \frac{4}{m} + \frac{6}{m^3} - \frac{8}{m^4} + \frac{2}{m^5} + \frac{4}{m^6} - \frac{6}{m^7} + \frac{4}{m^8} - \frac{1}{m^9}\right) \\ p_{5,m}^1 ={}&\frac{1}{m^4} \bigg (5 - \frac{5}{m} + \frac{10}{m^4} - \frac{15}{m^5} + \frac{5}{m^6} + \frac{10}{m^8} - \frac{20}{m^9} + \frac{15}{m^{10}} - \frac{5}{m^{11}} + \frac{5}{m^{12}} -\frac{10}{m^{13}} \\&\,\qquad + \frac{10}{m^{14}} - \frac{5}{m^{15}} + \frac{1}{m^{16}}\bigg ). \end{aligned}$$

Our result is different from the one in [20], where the frequency of games with one PSNE, but possibly also mixed strategy Nash equilibria, converges to $\exp (-1)$ as the number of strategies for at least two players goes to infinity.

**Fig. 2:**
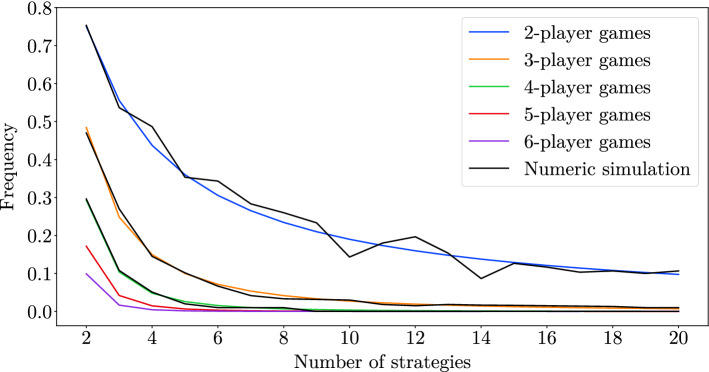
The frequency of randomly drawn games that have a unique PSNE

Figure 2 shows the frequency of randomly created games with a unique PSNE. For comparison, we show frequencies obtained by numerically sampling over 500 randomly created games with payoffs drawn from a normal distribution.

### Type B: Convergent Games with Multiple PSNEs

We can bound the frequency of convergent games with more than one PSNE from above:

#### Theorem 2

For $k_1<k_2$, we have $p_{n,m}^{k_1} > p_{n,m}^{k_2}$.

Theorems 1 and 2 imply that for every *k*, $p^k_{n,m}\rightarrow 0$ as the number of strategies or the number of players goes to infinity. We computed for 3-player, 2-strategy games that $p_{3,2}^1=\frac{1984}{4096}\approx 48.43\%$, $p_{3,2}^2=\frac{828}{4096}\approx 20.21\%$, $p_{3,2}^3=\frac{56}{4096}\approx 1.37\%$, $p_{3,2}^4=\frac{2}{4096}\approx 0.049\%$.

In two-player games, we can exactly state the frequency of games with *k* PSNEs.

#### Theorem 3

The frequency of 2-player, *m*-strategy convergent games with exactly *k* PSNEs in the ensemble is given by

$$\begin{aligned} p_{2,m}^k = \frac{2m-k}{m^{2k+2} (k-1)!} \left( \frac{m!}{(m-k)!}\right) ^2. \end{aligned}$$

for $k\le m$, and is otherwise 0.

The frequency of drawing a 2-player convergent game (Type A or Type B) is then given by $\sum _{k=1}^m p_{2,m}^k$, the frequency of Type B games only is $\sum _{k=2}^m p_{2,m}^k$. Numerical evidence shows that Type A games are more common than Type B games for $m=2,\dots ,9$, and less common for $m\ge 10$.

**Fig. 3:**
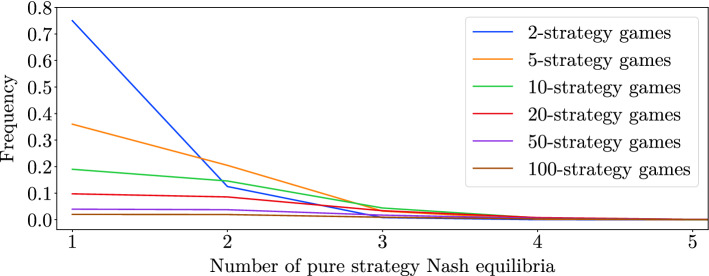
The frequency of randomly drawn convergent 2-player games that have a given number of PSNEs

**Fig. 4:**
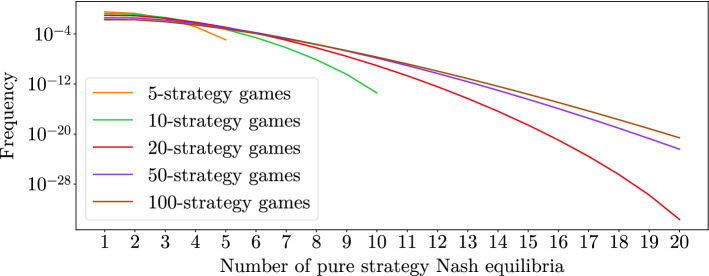
The frequency of randomly drawn convergent 2-player games that have a given number of PSNEs where the frequency is log-scaled

Figures 3 and 4 show the frequency of randomly drawn convergent 2-player games that have a given number of PSNEs.

## Conclusion

We have investigated the frequency of games that are convergent under a best-response dynamic, in which each player chooses their optimal pure strategy successively. Such games may either be perfectly predictable, if they have a unique PSNE, or have multiple PSNEs. We analytically computed the frequency of the first type by using a novel graph-theoretic approach for describing games, and showed that if we let the number of players or the number of strategies go to infinity, almost all games do not converge. We also showed that games with a higher number of PSNEs are less common than games with a smaller number of PSNEs. This calls the validity of simple models for complex scenarios into question. If a simple scenario is to be modelled, a Type A game could be the right approach. However, for complex scenarios, models based on Type A or Type B games can lead to misleading results. Instead, techniques from agent-based modelling, network theory, or Bayesian statistics could be employed.

For 2-player games, we gave an exact formula for the frequency of games with a given number of PSNEs, and highlight that for less than 10 strategies, games with a unique PSNE are more common than convergent games with multiple PSNEs, otherwise less common.

We believe that our graph-theoretic approach can generally be very useful to understand complicated games. Extensions of this work would include finding the analytical frequency of multi-player games with multiple pure Nash equilibria or with mixed Nash equilibria.

## Proofs

### Proof of Theorem 1

Consider the full *n*-partite graph for an *n*-player, *m*-strategy game. We order the nodes in the following way: $s_{-i}<s_{-j}$ for different players *i* and *j*, if and only if $i<j$, and for the same player *i*, $s_{-i}<s'_{-i}$ under lexicographical ordering. Denote this full *n*-partite graph by $G^\text {f}=(V^\text {f},E^\text {f})$, where *V* is the set of vertices and *E* is the set of edges.

The Laplacian matrix of a graph $G=(V,E)$ without multiple edges and self-loops is defined as the square matrix with side length |*V*| and

$$\begin{aligned} \left( L(G)\right) _{ij} = {\left\{ \begin{array}{ll} \delta ^+(i) &{} \text {if } i = j \\ -1 &{} \text {if } i\ne j, \,(i,j)\in E \\ 0 &{} \text {if } i\ne j, \,(i,j)\not \in E \end{array}\right. } \end{aligned}$$

where $\delta ^+(i)$ is the out-degree of a node *i*. For $G^\text {f}$ described above, the Laplacian matrix takes the following form: 
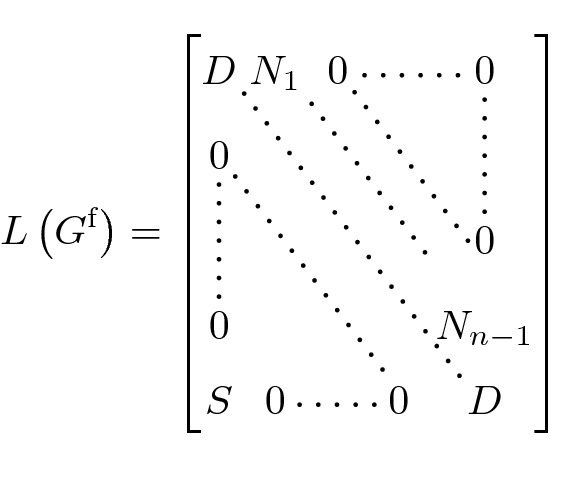


where $D,S,N_1,\dots ,N_{n-1}$ are square matrices with side length $m^{n-1}$ defined as follows:

- $D=\text {diag}(m)$ is a diagonal matrix with *m*’s on the diagonal
- $N_k=\text {diag}\left( K_1,\dots ,K_{m^{k-1}}\right)$ is a blockmatrix with blockmatrices $K_l$ on the diagonal, where each $K_l$ has side length $m^{n-l}$ and consists of $m^2$ diagonal matrices $\text {diag}(-1)$, each with side length $m^{n-l-1}$.
- *S* is more irregular,
  $$\begin{aligned} (S)_{ij} = {\left\{ \begin{array}{ll} -1 &{} \text {if } \left( i \mod m^{n-2}\right) = \left\lfloor \frac{j-1}{m}\right\rfloor \\ 0 &{} \text {otherwise}. \end{array}\right. } \end{aligned}$$

**Fig. 5:**
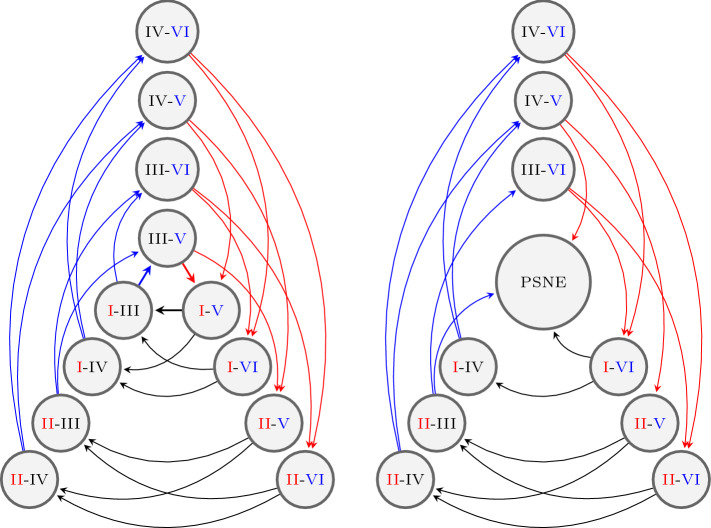
For 3-player, 2-strategy games the full graph $G^\text {f}$ on the left and the condensed graph $G^\text {c}$ on the right

For instance, in the case of 3-player, 2-strategy games, the Laplacian matrix corresponding to Fig. 5 (left) is given by

$$\begin{aligned} L\left( G^\text {f}\right) = \left[ \begin{array}{cccc|cccc|cccc} 2 &{} 0 &{} 0 &{} 0 &{} -1 &{} 0 &{} -1 &{} 0 &{} 0 &{} 0 &{} 0 &{} 0 \\ 0 &{} 2 &{} 0 &{} 0 &{} 0 &{} -1 &{} 0 &{} -1 &{} 0 &{} 0 &{} 0 &{} 0 \\ 0 &{} 0 &{} 2 &{} 0 &{} -1 &{} 0 &{} -1 &{} 0 &{} 0 &{} 0 &{} 0 &{} 0 \\ 0 &{} 0 &{} 0 &{} 2 &{} 0 &{} -1 &{} 0 &{} -1 &{} 0 &{} 0 &{} 0 &{} 0 \\ \hline 0 &{} 0 &{} 0 &{} 0 &{} 2 &{} 0 &{} 0 &{} 0 &{} -1 &{} -1 &{} 0 &{} 0 \\ 0 &{} 0 &{} 0 &{} 0 &{} 0 &{} 2 &{} 0 &{} 0 &{} -1 &{} -1 &{} 0 &{} 0 \\ 0 &{} 0 &{} 0 &{} 0 &{} 0 &{} 0 &{} 2 &{} 0 &{} 0 &{} 0 &{} -1 &{} -1 \\ 0 &{} 0 &{} 0 &{} 0 &{} 0 &{} 0 &{} 0 &{} 2 &{} 0 &{} 0 &{} -1 &{} -1 \\ \hline -1 &{} -1 &{} 0 &{} 0 &{} 0 &{} 0 &{} 0 &{} 0 &{} 2 &{} 0 &{} 0 &{} 0 \\ 0 &{} 0 &{} -1 &{} -1 &{} 0 &{} 0 &{} 0 &{} 0 &{} 0 &{} 2 &{} 0 &{} 0 \\ -1 &{} -1 &{} 0 &{} 0 &{} 0 &{} 0 &{} 0 &{} 0 &{} 0 &{} 0 &{} 2 &{} 0 \\ 0 &{} 0 &{} -1 &{} -1 &{} 0 &{} 0 &{} 0 &{} 0 &{} 0 &{} 0 &{} 0 &{} 2 \end{array}\right] . \end{aligned}$$

There are $m^n$ ways to choose the first PSNE, each fixing *n* nodes. Without loss of generality, we choose the nodes where each player chooses their first strategy. We condense these *n* nodes to a single node representing the PSNE, see Fig 5. The PSNE-node has an in-degree of $n(m-1)$; we delete all outgoing edges. The resulting $(n+1)$-partite graph consists of $nm^{n-1}-(n-1)$ nodes and will be denoted by $G^{\text {c}}=(V^\text {c},E^\text {c})$, where $V^\text {c}$ is the set of vertices and $E^\text {c}$ is the set of edges. All nodes except the PSNE-node are free.

We apply Kirchhoff’s theorem to $G^{\text {c}}$ to get the number of spanning trees. This guarantees that the game converges under clockwork best-response dynamics. Kirchhoff’s theorem (applied to our problem) states that the number of spanning trees is the determinant of the Laplacian matrix of $G^\text {c}$ with the first row and column deleted, which corresponds to the PSNE-node. For a quadratic matrix A with side length *n*, we define ${\widetilde{A}}$ to be the quadratic matrix with side length $(n-1)$ obtained from *A* by deleting the first row and column.

For a general blockmatrix $$K=\left( \begin{matrix} A &{} B \\ C &{} D\end{matrix}\right) $$, provided that *A* is invertible, we have

$$\begin{aligned} \det K = \det \left( D-C A^{-1} B\right) \det A. \end{aligned}$$

Applying this identity iteratively to $\widetilde{L\left( G^\text {c}\right) }$ yields

$$\begin{aligned} \det \widetilde{L\left( G^\text {c}\right) } = m^{\left( m^{n-1}-1\right) (n-1)} \cdot \det \left( {\widetilde{D}} - \frac{1}{m^{n-1}}\cdot {\widetilde{S}}\cdot \prod _{i=1}^{n-1} \widetilde{N_i}\right) . \end{aligned}$$

The matrix ${\widetilde{S}}\cdot \prod _i \widetilde{N_i}$ is given by

$$\begin{aligned} \left( {\widetilde{S}}\cdot \prod _i \widetilde{N_i}\right) _{ij} = m - \mathbbm {1}_{\left[ 1,m^{n-\delta (i)}-1\right] }(j) \end{aligned}$$

where

$$\begin{aligned} \delta (i) := {{\,\mathrm{arg\,min}\,}}_{p\in [1,n-1]} \left( \min _{k\in [1,m^p]}\left( \left| i-km^{n-p-1}\right| \right) \right) . \end{aligned}$$

We simplify the matrix ${\widetilde{D}} - \frac{1}{m^{n-1}}\cdot {\widetilde{S}}\cdot \prod _{i=1}^{n-1} \widetilde{N_i}$ by elementary row- and column-operations to obtain a matrix *A* by the following algorithm: 
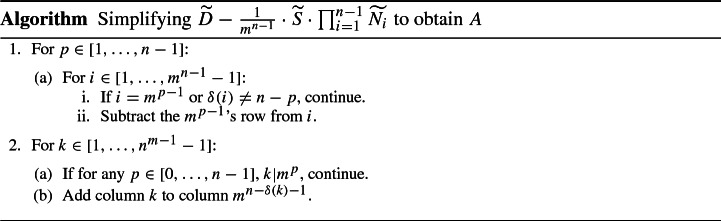


The determinant of the matrix *A* can be written as

$$\begin{aligned} \det A = m^{m^{m-1}-n} \cdot \det {\widehat{A}} \end{aligned}$$

for a matrix ${\widehat{A}}$ with side length $(n-1)$ and given by

$$\begin{aligned} \left( {\widehat{A}}\right) _{ij} = {\left\{ \begin{array}{ll} m+\frac{m-1}{m^{n-1}}-\frac{m-1}{m^{i-1}} &{} i=j \\ \frac{m-1}{m^{n-i+j-1}}-\frac{m-1}{m^{j-1}} &{} i>j \\ -\frac{m-1}{m^{j-1}} &{} i<j \end{array}\right. } \end{aligned}$$

Adding the *i*-th column multiplied by $\left( -\frac{1}{m}\right)$ to the $(i+1)$-th column for $i=n-2,n-3,\dots ,1$, we get a matrix of the following form 
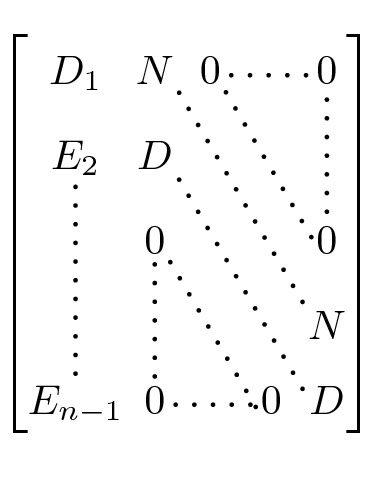


where

$$\begin{aligned} D_1 ={}&\frac{m-1}{m^{n-1}}+1 \\ E_i ={}&\frac{m-1}{m^{n-i}} - (m-1) \\ D ={}&m \\ N ={}&-\frac{m-1}{m^n}-1. \end{aligned}$$

To eliminate the *N* entries on the upper diagonal, we add the *i*-th row multiplied by

$$\begin{aligned} F := -\frac{N}{D} = \frac{\frac{m-1}{m^n}+1}{m} \end{aligned}$$

to the $(i-1)$-th row for $i=n-1,\dots ,2$. Then, the matrix is lower-triangular and the $D_1$ entry is given by

$$\begin{aligned} \widetilde{D_1} ={}&D_1+F\cdot E_2+\cdots +F^{n-2}\cdot E_{n-1} \\ ={}&D_1+\sum _{i=0}^{n-3} F^{i+1}\cdot E_{i+2} \\ ={}&D_1+ \sum _{i=0}^{n-3} F^{i+1} \left( \frac{m-1}{m^{n-i-2}}\right) - \sum _{i=0}^{n-3} F^{i+1} (m-1) \\ ={}&D_1 + F\left( \frac{m-1}{m^{n-2}}\right) \sum _{i=0}^{n-3} \left( Fm\right) ^i - F(m-1) \sum _{i=0}^{n-3} F^i \\ ={}&D_1 + F\left( \frac{m-1}{m^{n-2}}\right) \left( \frac{(Fm)^{n-2}-1}{Fm-1}\right) - F(m-1)\left( \frac{F^{n-2}-1}{F-1}\right) \\ ={}&m(r-1)+m\left( r^{n-1}-r\right) -\frac{r(m-1)}{r-m} \left( \left( \frac{r}{m}\right) ^{n-2}-1\right) +1 \end{aligned}$$

where $r:=\frac{m-1}{m^n}+1$, and then

$$\begin{aligned} \det {\widehat{A}} ={}&m^{n-2}\cdot \widetilde{D_1}. \end{aligned}$$

Finally, the frequency of games with exactly one PSNE is given by

$$\begin{aligned} p^1_{n,m} = \frac{m^n}{m^{nm^{n-1}}} \det \widetilde{L\left( G^\text {c}\right) } = \frac{1}{m^{m^{n-1}-1}} \det A = \frac{1}{m^{n-1}} \det {\widehat{A}} = \frac{1}{m}\widetilde{D_1} \end{aligned}$$

where we have multiplied by the number of possible positions of the PSNE and divided by the total number of possible arrangements. This completes the proof.

### Proof of Theorem 2

Consider a full *n*-partite graph and assign *k* PSNEs, thereby fixing the outgoing edges of *kn* nodes. We show that the number of possible realizations as a game decreases, when adding another PSNE.

The number of ways we can add another PSNE (which is, in general, very complicated to compute) is bounded from above by $\left( m^{n-1}-k\right) m=m^n-km$, which is because there are $m^{n-1}-k$ free nodes for each player, each free node has an out-degree of *m*, and fixing two nodes of an *n*-cycle fixes the remaining ones. However, adding a PSNE decreases the number of possible realizations as a game by a factor of $m^n-1$, because the *n* nodes may not form a cycle.

Induction over the number of added PSNEs completes the proof.

### Proof of Theorem 3

It was shown in Austin [2] that the number of chromatic digraphs with *m* nodes of each type, where each node has an out-degree one, and with a cycle of length 2*k*, $1\le k\le m$, is

$$\begin{aligned} (2m-k) \left( m^{m-k-1}\right) ^2 \left( \frac{m!}{(m-k)!}\right) ^2. \end{aligned}$$

Factoring out the number of ways to arrange *k* vertices on a cycle ($$(k+1)!$$) and the total number of possible arrangements ($$m^{2m})$$, we get

$$\begin{aligned} p_{2,m}^k = \frac{2m-k}{m^{2k+2} (k-1)!} \left( \frac{m!}{(m-k)!}\right) ^2. \end{aligned}$$

This was given in Pangallo et al. [17] as a recursively defined formula.
